# Efficacy of a 3-month lifestyle intervention program using a Japanese-style healthy plate on body weight in overweight and obese diabetic Japanese subjects: a randomized controlled trial

**DOI:** 10.1186/1475-2891-13-108

**Published:** 2014-11-24

**Authors:** Keiko Yamauchi, Tomomi Katayama, Takahiro Yamauchi, Kazuhiko Kotani, Kokoro Tsuzaki, Kaoru Takahashi, Naoki Sakane

**Affiliations:** Department of Nutritional Science, Nagoya University of Arts and Sciences, Aichi, Japan; Takarazuka University School of Nursing, Osaka, Japan; Nursing Department, Gamagori City Hospital, Aichi, Japan; Division of Preventive Medicine, Clinical Research Institute, National Hospital Organization Kyoto Medical Center, Kyoto, Japan; Hyogo Health Service Association, Hyogo, Japan

**Keywords:** Portion control plate, Weight loss, Diabetes, Obesity

## Abstract

**Background and objectives:**

The portion size of food is a determinant of energy intake, linking with obese traits. A healthy plate for portion control has recently been made in a Japanese style. The aim of the current study was to assess the efficacy of a lifestyle intervention program using the Japanese-style healthy plate on weight reduction in overweight and obese diabetic Japanese subjects.

**Methods:**

We randomized overweight and obese diabetic subjects (n = 19, 10 women) into an intervention group including educational classes on lifestyle modification incorporating the healthy plate (n = 10) or a waiting-list control group (n = 9). The intervention period was three months, and the educational classes using the healthy plate were conducted monthly in a group session for the intervention group. The body weight, blood glycemic and metabolic measures, and psychosocial variables were measured at the baseline and after the 3-month intervention in both groups. The impression of the intervention was interviewed using a structured questionnaire.

**Results:**

There was one drop-out in the control group. No adverse events were reported in the groups. Subjects in the intervention group had a greater weight change from baseline to the end of the 3-month intervention period (-3.7 +/- 2.5 [SD] kg in the intervention group vs. -0.1 +/- 1.4 kg in the control group, P = 0.002). Most subjects recorded that the use of a healthy plate could be recommended to other people.

**Conclusions:**

The lifestyle intervention program using the Japanese-style healthy plate, which was developed for portion control, may effectively reduce body weight in overweight and obese diabetic subjects in Japan. Further studies are needed to establish the efficacy of this methodology on weight management.

## Background

The obesity epidemic has become a worldwide phenomenon in recent years [1, 2]. In Japan, the trend is the same [3]. Obesity is associated with an increased risk of morbidity and mortality [2]. Lifestyle modification, including diets, is the cornerstone for the prevention and improvement of obesity as a preventative method [4]. The portion size of food has a significant influence on energy intake, linking with obesity traits [5].

A plate is a flat and round dish that Japanese people often use at mealtimes; accordingly, a plate can be used as a tool for dietary intervention. Indeed plates for portion control have been used in previous studies [6, 7]; however, the plates were made for western-style dishes. With respect to the development of metabolic disorders, ethnic/racial differences are known (i.e., the difference in the level of insulin secretion between Japanese and Caucasian people) [8, 9]. Differences in diet culture are widely recognized between countries. One of the latest topics debated of this field is a Japanese meal, especially a traditional meal, as a tool for management of metabolic disorders. [10, 11]. The typical Japanese meal consists of a bowl of rice (gohan), a bowl of miso soup (miso shiru), pickled vegetables (tsukemono) and fish or meat. Rice is the staple food, and several kinds of noodles (udon, soba and ramen) are also popular as light meals. As an island nation, the Japanese people frequently eat seafood. A wide variety of fish, squid, octopus, eel, and shellfish appear in all kinds of dishes from sushi to tempura [12, 13].

This background can require a healthy plate for portion control according to the Japanese food style in consideration of an intervention with portion control for the Japanese population. Thus, a Japanese-style healthy plate has recently been developed. The aim of the current study was to assess the efficacy of a lifestyle intervention program using the Japanese-style healthy plate on weight reduction in overweight and obese diabetic Japanese subjects.

## Methods

### Study design

**Figure 1:**
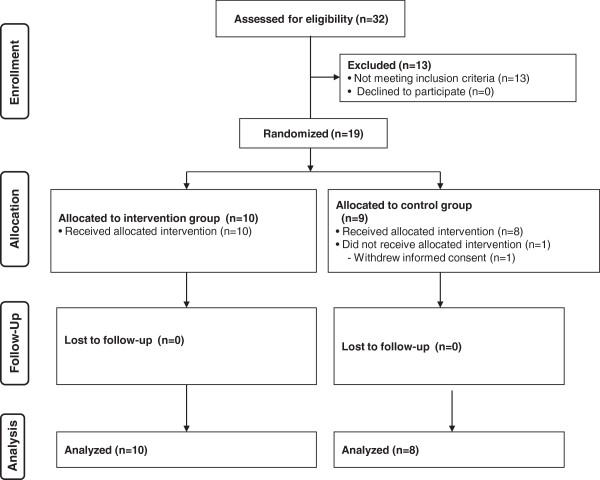
**CONSORT flow diagram.**

The study design is according to the recommendations of the CONSORT statement for randomized trials of non-pharmacologic treatment [14] (Figure 1). This study was an unmasked, randomized controlled trial. We randomized overweight and obese diabetic subjects into an intervention group including educational classes on lifestyle modification incorporating the healthy plate or a waiting-list control group. The randomization was stratified by sex using sealed envelopes by an independent statistician, because sex is considered a strong factor affecting weight changes in intervention trials [15–17].

Potential subjects were recruited from 15 clinics in Toyohashi city, Aichi prefecture, Japan. These clinics were in primary health care settings to maintain the homogeneity of the studied subjects. Recruitment started in March 2012 and was completed in December 2012. Those who met one of the following criteria were recruited: 1) aged 20-70 years, 2) type 2 diabetes mellitus or 3) body mass index (BMI) ≥24 kg/m^2^ (the overweight level was defined based on previous studies [18–21]). Those with the following criteria were excluded 1) diabetes mellitus under insulin therapy, 2) dementia or 3) chronic renal failure. The study was approved by the Ethics Committee of the National Hospital Organization Kyoto Medical Center and Nagoya University of Arts and Sciences. All subjects gave their written informed consent.

### Intervention group

**Figure 2:**
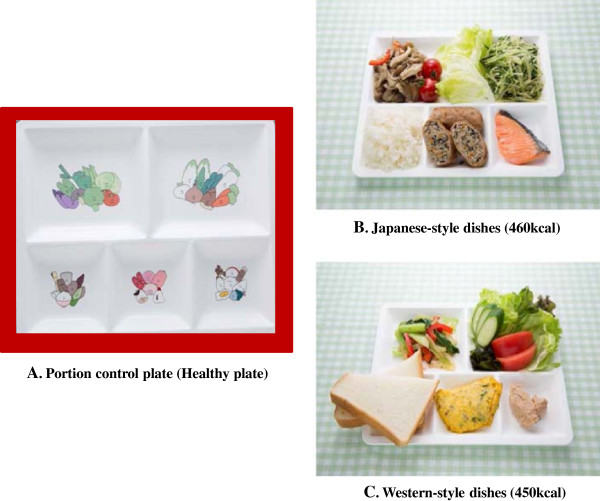
**Portion control plate (Healthy plate) and examples.**

The schedule and contents of intervention in the intervention and control groups are listed in Table 1. These lectures were provided to all participants in a public healthcare facility near the clinics. At baseline, a lecture titled, “What is diabetes and its complications?” by diabetic educators and, “Eating healthy with diabetes” by registered dieticians (RD) was offered to the subjects. In educational classes, a specific lecture of about 3.5 hours was given monthly during the 3-month intervention period. The main topics of each lecture were as follows: (part 1) “Tips to reduce body weight with the healthy plate (Healthy Plate^®^; HYPK, Co., Ltd., Aichi, Japan), What is portion control?”, (2) “Tips to maintain weight using the healthy plate”, and (3) “Tips to prevent weight gain when eating out”. After each lecture, there was a group discussion with the RD. In particular, over time, the RD reviewed food choices, consistency and timing of meals, meal plans and appropriate use of snacks, in addition to portion control (i.e. how to use the healthy plate). The healthy plate is made of pottery and porcelain with colorful print dividing it into four sections (Figure 2). It is 27.4 cm long, 21.0 cm wide and 2.5 cm deep. Three-sixths (one-half) of the plate was labeled “vegetables”, one-sixth was labeled “rice, bread and noodles” and two-sixth were labeled “fish, meat, chicken and nuts”. One section was similar to 1 unit (= 80 kcal) of the food exchange list. The subjects were instructed to use the plate for their largest meal of the day and encouraged to use the plate for all meals.

**Table 1:** **Schedule of intervention and assessment in both arms**

Schedule order	Time	Contents	Intervention arm	Control arm
Baseline assessment	9:00 am-0:30 pm (3.5 hours)	Lecture: “What is diabetes?” by diabetes educators and “Eating healthy with diabetes” by dietitians. Baseline data collection.	Done	Done
Educational class-Part 1	10:00 am-1:30 pm (3.5 hours)	Ice breaking: buffet game using 80-kcal food cards. Lecture: “Tips to reduce body weight with a healthy plate” and “What is carbohydrate control?” by dietitians. Lunch using the healthy plate. Discussion.	Done	Not done
Educational class-Part 2	10:00 am-1:30 pm (3.5 hours)	Ice breaking: game of sweets tournament. Lecture: “Tips to maintain weight using a health plate” by dietitians. Lunch using the healthy plate. Discussion.	Done	Not done
Educational class--Part 3	10:00 am-1:30 pm (3.5 hours)	Ice breaking: experience of a virtual Japanese-style pub. Lecture: “Tips to prevent weight gain when eating out” by dietitians. Lunch using the healthy plate. Discussion.	Done	Not done
Final assessment	10:00 am-0:00 pm (2 hours)	Final data collection.	Done	Done

### Control group

At baseline, a lecture titled, “What is diabetes and its complications?” by diabetic educators and, “Eating healthy with diabetes” by a RD was offered to the subjects, similar to the intervention group. After this initial lecture, the subjects in the control group were advised to continue their routine health care at each clinic. The control group received standard care including dietary advice from a doctor.

### Measurements

All measurements were conducted in the same order for all subjects in the intervention and control groups at the baseline and after the 3-month intervention period. We instructed the subjects not to participate in vigorous physical activity or to consume alcohol within the 24 hours prior to the measurements. Body weight was measured to the nearest 0.1 kg using a digital scale (TBF-551; Tanita Company, Tokyo, Japan) and height was measured once to the nearest 0.1 cm using a wall-mounted stadiometer (YG-200; Yagami Company, Nagoya, Japan). For both measurements, the subjects wore only underwear and were barefooted and they had been fasting for at least 8 hours. The BMI was calculated as weight (in kilograms) divided by height (in meters) squared. The waist circumference was measured directly on the skin surface at the level of the umbilicus in a standing position [22]. The abdominal circumference measurements were taken in duplicate to the nearest 0.1 cm, and the averaged value was used for the analysis.

The following clinical data, blood pressure, serum lipids (total cholesterol, low-density lipoprotein [LDL]-cholesterol, high-density lipoprotein [HDL]-cholesterol, triglycerides), creatinine (estimated glomerular filtration rate as calculated by creatinine), aspartate aminotransferase (AST), alanine aminotransferase (ALT), γ-glutamyltransferase (γGT), plasma glucose and hemoglobin A1c (HbA1c), were measured at a central laboratory (Okazaki Medical Association Public Health Center, Aichi, Japan).

The following psychosocial variables were also assessed. The levels of appetite, dietary satisfaction and distress related to the diet were recorded using 100 mm visual analogue scales (VAS). An integrated appetite score was especially calculated as the score: VAS hunger + VAS prospective consumption + (100-VAS gastric fullness)/3 [23]. The desire levels to eat sweet, salty, spicy and rich foods were also recorded using the VAS. A revised version of the three-factor eating questionnaire 18 (TFEQ-R18) measures 3 aspects of eating behaviors, such as cognitive restraint, uncontrolled eating and emotional eating [24, 25]. The profile of mood states (POMS) test in a brief version is composed of 30 questions with a 5-point scale about the current mood state, which is classified into “tension-anxiety”, “depression-dejection”, “anger-hostility”, “vigor”, “fatigue” and “confusion” subscales [26, 27]. A low POMS score indicates a better mood state, except for the “vigor” factor.

Information on the use of anti-diabetic agents was also recorded during the intervention. Participants were asked “How often did you use the healthy plate per day during the intervention?” at the end of the study. Responses were classified as one, two, and three times per day. We did not monitor the use of the healthy plate every day during the intervention. After the intervention, a trained researcher independently interviewed each subject in the intervention group regarding their impression of the intervention, including satisfaction with the intervention, using a structured questionnaire.

Dietary intake and walking steps were measured only in the intervention group. Dietary intake was assessed using standardized software for population-based surveys and nutrition counseling in Japan (EIYO-KUN v.6.0, developed by Shikoku University Nutrition Database) based on the Standard Tables of Food Composition in Japan [28, 29]. Steps were counted using a pedometer (Omron HJA-307IT; Omron Corporation, Kyoto, Japan). Daily steps were averaged over 7 days.

### Outcomes

The main outcomes were the changes of obesity traits, including weight and BMI, and blood tests during the intervention. The secondary outcomes were the changes of psychosocial variables.

### Sample-size estimation

A sample of 18 subjects was calculated calculation based on detecting a difference of 3 kg and a standard deviation (SD) of 2 kg in weight loss at the 3 month follow-up between intervention and control group, with 80% power and 5% significance. The difference (plus SD) was based on a previous study that examined weight changes with a lifestyle modification, similar to the current study [19]. The sample size was calculated using StatMate software.

### Statistical methods

Paired t-tests were used to analyze the differences in the data at the baseline and the 3-month changes, and a two-way (group and duration factor) ANOVA was used to analyze the differences in the data between the groups. We conducted analyses by excluding subjects with missing data (complete case analysis). Two-sided p <0.05 was considered significant.

## Results

Figure 1 shows a flow diagram of the study subjects. The intervention and control groups had similar clinical characteristics (Table 2). While there was one drop-out in the control group, the subjects in the intervention group completed the study. No adverse events were reported in the groups.

**Table 2:** **Baseline and 3-month data of clinical characteristics**

Variables	Intervention group (n = 10)				Control group (n = 8)				P^2^
	Baseline	3 months	Difference	P^1^	Baseline	3 months	Difference	P^1^	
Age, years	55.8 (10.4)	-		-	59.0 (11.9)			-	-
Male, % (number)	50% (5)	-		-	50% (4)			-	-
Weight, kg	71.3 (17.0)	67.6 (15.6)	-3.7 (2.5)	0.001	72.9 (9.6)	72.8 (9.7)	-0.1 (1.4)	0.813	0.002
BMI, kg/m^2^	27.6 (3.8)	26.2 (3.6)	-1.4 (0.8)	<0.001	28.4 (2.4)	28.3 (2.2)	-0.1 (0.5)	0.756	0.001
Waist circumference, cm	93.6 (10.6)	89.3 (11.4)	-4.2 (2.7)	0.001	92.9 (7.9)	93.5 (7.9)	0.6 (1.6)	0.321	<0.001
SBP, mmHg	133.7 (8.4)	119.8 (11.5)	-13.9 (13.6)	0.010	127.1 (8.3)	129.1 (11.3)	2.0 (9.0)	0.549	0.012
DBP, mmHg	79.5 (8.5)	72.1 (10.8)	-7.4 (8.2)	0.019	71.0 (4.5)	74.3 (6.5)	3.3 (5.2)	0.119	0.006
Plasma glucose, mmol/L	7.1 (1.7)	5.9 (1.6)	-1.1 (1.9)	0.089	7.9 (3.1)	6.8 (2.7)	-1.2 (3.8)	0.410	0.981
HbA1c, %	7.6 (1.2)	6.7 (0.8)	-0.9 (0.9)	0.008	8.0 (1.4)	7.3 (1.0)	-0.7 (1.1)	0.112	0.606
Anti-diabetic agents, number	2.5 (0.9)	2.2 (0.8)	-0.3 (0.5)	0.081	3.3 (0.9)	3.3 (0.9)	0.0 (0.0)	1.000	0.153
Total cholesterol, mmol/L	4.47 (0.88)	4.55 (0.80)	0.07 (16.2)	0.623	4.81 (0.78)	4.81 (0.57)	-0.02 (0.53)	0.935	0.732
LDL-cholesterol, mmol/L	2.64 (0.65)	2.77 (0.62)	0.12 (0.43)	0.408	2.87 (0.65)	2.77 (0.65)	-0.10 (0.36)	0.509	0.285
HDL-cholesterol, mmol/L	1.29 (0.36)	1.37 (0.36)	0.09 (0.12)	0.056	1.45 (0.39)	1.50 (0.36)	0.04 (0.13)	0.407	0.462
Triglycerides, mmol/L	1.19 (0.55)	0.88 (0.29)	-0.30 (0.38)	0.036	1.16 (0.73)	1.37 (0.75)	0.20 (0.45)	0.255	0.028
Serum creatinine, mmol/L	61.0 (15.9)	62.8 (15.0)	2.7 (2.7)	0.035	60.1 (15.9)	58.3 (16.8)	-1.8 (7.1)	0.475	0.151
eGFR, Unit	86 (23)	81 (19)	-4.6 (6.8)	0.061	85 (25)	85 (22)	0.4 (9.8)	0.914	0.220
AST, U/L	38 (28)	28 (13)	-9.4 (18.8)	0.149	25 (11)	23 (8)	-1.25 (4.7)	0.480	0.216
ALT, U/L	56 (51)	36 (23)	-19.6 (32.8)	0.091	32 (25)	32 (28)	0.3 (7.3)	0.926	0.092
γGT, U/L	35 (18)	31 (31)	-3.4 (26.0)	0.688	36 (15)	35 (15)	-1.5 (2.1)	0.080	0.840

BMI: body mass index, SBP: systolic blood pressure, DBP: diastolic blood pressure, LDL: low-density lipoprotein, HDL: high-density lipoprotein, eGFR: estimated glomerular filtration rate, AST: aspartate aminotransferase, ALT: alanine aminotransferase, γGT: γ-glutamyl transferase. Values are the means (standard deviations) or numbers. P^1^: difference within the groups, P^2^: difference between the groups.

As shown in Table 2, those of the intervention group had a greater weight change from baseline to the 3-month intervention period (-3.7 ± 2.5 [SD] kg in the intervention group *vs*. -0.1 ± 1.4 kg in the control group, p = 0.002). The BMI and waist circumference showed the same trend as weight. Subjects in the intervention group had a greater reduction in the level of blood pressure or serum triglycerides from baseline to the end of the 3-month intervention period. The HbA1c level was reduced after intervention in the intervention group, although the changes in HbA1c levels did not significantly differ between the intervention and control groups. The other variables did not show any significant difference between the intervention and control groups.

As shown in Table 3, there were no relevant changes in the psychosocial variables from the baseline to the end of the 3-month intervention period. Uncontrolled eating in the TFEQ-R18 differed between the groups, but a reduction was seen in the control group; therefore, the changes did not seem to be relevant.

**Table 3:** **Baseline and 3-month scores of psychosocial characteristics**

Variables	Intervention group (n = 10)				Control group (n = 8)				P^2^
	Baseline	3 months	Difference	P^1^	Baseline	3 months	Difference	P^1^	
TFEQ-R18									
Uncontrolled eating	17.1 (5.4)	17.3 (5.2)	0.2 (4.7)	0.895	24.9 (3.3)	18.9 (3.0)	-5.7 (5.7)	0.038	0.044
Cognitive restraint	18.8 (2.7)	20.7 (4.3)	1.9 (4.0)	0.169	16.0 (0.8)	17.4 (2.6)	1.4 (3.1)	0.269	0.789
Emotional eating	9.0 (2.1)	8.3 (3.1)	-0.4 (2.4)	0.606	6.6 (2.4)	8.4 (1.9)	1.9 (3.9)	0.258	0.208
Appetite score, mm	3.5 (3.6)	3.4 (2.2)	-0.2 (4.2)	0.913	2.2 (1.9)	2.2 (1.9)	0.2 (2.8)	0.991	0.861
Dietary satisfaction, mm	4.6 (2.8)	4.1 (2.5)	-0.6 (3.2)	0.593	6.1 (3.3)	6.5 (2.5)	0.6 (3.1)	0.745	0.506
Dietary distress, mm	4.1 (2.3)	2.8 (2.9)	-1.3 (3.4)	0.265	7.6 (1.3)	5.8 (1.1)	-1.7 (2.5)	0.068	0.804
Desire to eat									
Sweet food	6.1 (2.6)	5.6 (3.0)	-0.4 (3.1)	0.666	2.8 (2.1)	3.7 (2.2)	0.2 (1.4)	0.353	0.613
Salty food	7.2 (1.6)	7.0 (2.9)	-0.2 (2.1)	0.727	5.7 (2.1)	6.6 (1.6)	0.9 (2.5)	0.308	0.438
Spicy food	7.7 (1.7)	7.0 (2.6)	-0.7 (2.0)	0.313	5.7 (1.7)	6.6 (1.6)	0.5 (2.0)	0.311	0.301
Rich food	8.1 (1.7)	7.3 (2.8)	-0.9 (2.5)	0.317	6.8 (2.2)	6.5 (2.2)	-0.2 (2.6)	0.767	0.671
POMS									
Tension-anxiety	9.4 (2.5)	8.1 (3.5)	-1.3 (4.1)	0.340	10.9 (3.4)	9.9 (2.5)	-1.0 (3.9)	0.520	0.880
Depression-dejection	7.1 (2.2)	6.9 (2.7)	-0.2 (3.7)	0.869	8.6 (2.5)	9.0 (2.1)	0.6 (4.2)	0.754	0.727
Anger-hostility	7.4 (2.1)	7.5 (2.8)	0.1 (3.8)	0.936	9.7 (1.9)	9.9 (2.1)	-0.4 (2.4)	0.869	0.763
Vigor	12.0 (2.2)	14.2 (3.4)	2.2 (4.1)	0.122	12.1 (3.0)	12.3 (2.9)	0.8 (2.8)	0.911	0.450
Fatigue	7.9 (1.6)	8.6 (3.9)	0.7 (4.4)	0.625	12.4 (4.0)	10.4 (1.9)	-0.8 (2.5)	0.243	0.414
Confusion	8.9 (1.1)	9.4 (2.4)	0.5 (2.6)	0.563	10.7 (2.5)	9.9 (2.5)	-1.2 (4.9)	0.597	0.497

TFEQ: three-factor eating questionnaire, POMS: profile of mood states. Values are the means (standard deviations). P^1^: difference within the groups, P^2^: difference between the groups.

In the intervention group, 86% of subjects recorded that the intervention, especially as provided by the RD, was helpful for weight reduction, and 63% recorded that they would like to recommend the healthy plate to other people, such as family members and friends. Twenty-five percent, 50% and 25% of the subjects reported using the plate for one meal per day, two and three meals per day, respectively. Seventy-five percent of subjects reported that they would continue to use the healthy plate after the study.

Total energy intake (1,796.3 ± 352.7 kcal vs. 1,312.6 ± 266.0 kcal; *P* < 0.001), carbohydrate intake (250.4 ± 43.5 g vs. 157.5 ± 40.2 g; *P* < 0.001), and protein intake (75.1 ± 24.0 g vs. 60.0 ± 13.0 g; *P* = 0.046) were significantly decreased after the 3-month intervention in the intervention group. However, there was no difference in fat intake (52.4 ± 10.5 g vs. 48.5 ± 8.7 g; *P* = 0.313) or fiber intake (14.1 ± 3.2 g vs. 14.8 ± 4.0 g; *P* = 0.701) between the baseline and after the 3-month intervention. Mean daily steps in the intervention group did not change from the baseline to after the 3-month intervention in the intervention group (6,873 ± 2,598 steps vs. 6,272 ± 2748 steps, respectively; *P* = 0.526).

## Discussion

### Main findings

This study showed that when compared to the control group, the lifestyle intervention program using the Japanese-style healthy plate for portion control significantly reduced body weight, concomitant with the BMI and waist circumference, in overweight and obese diabetic Japanese subjects. The glycemic control variables were not clearly altered in the intervention. The intervention program conducted largely featured the use of a Japanese-style healthy plate. This plate can be easily understood and accepted by the Japanese, leading to good portion control. Thus, it can be suggested that the use of the Japanese-style healthy plate may effectively control obesity traits in overweight and obese subjects in Japan. This Japanese-style healthy plate may be used as a future anti-obesity strategy in Japan.

The intervention using the Japanese-style healthy plate also significantly reduced the levels of blood pressure and serum triglycerides. These variables are components of obesity-related disorders and metabolic syndrome (MetS) [30]. Changes in lifestyles, including healthy dietary regimens, should be the first line of therapy against the MetS [31]. These variables may be improved through reduced insulin resistance following weight loss in obesity [32]. The reduction of HbA1c was also observed in the intervention group. Although the changes in HbA1c did not clearly differ compared to those of the control groups, we think that the HbA1c may also be reduced through the weight loss-related pathologic improvement [31, 33]. Given these data, although we focused on only a Japanese-style healthy plate as an anti-obesity tool in this study, the use of the plates might be further expanded as a possible intervention tool for multi-components associated with obesity in the future.

The intervention in the current study did not influence the psychosocial variables. On the other hand, most subjects in the intervention group reported that the intervention using the healthy plate was helpful for weight control. The results at least indicate that the intervention could not cause negative mood states, and rather, appeared to suggest favorable aspects of the intervention.

### Limitations

The limitations of the current study were the small sample size and short intervention period. Careful attention should also be paid on interpreting the results due to the insufficiency of statistical power with the small sample size [34]. Several randomized, controlled trials on weight management have employed at least a 3-month intervention [35, 36]. From the perspective of weight maintenance, there is an idea that the duration of dietary intervention had better be at least six months or one year. Future studies with a larger sample size and longer intervention should be performed.

There was a methodological limitation in that the frequency of using the healthy plate was asked only at the end of the study period in the intervention group. This can rely on recall information, and so careful attention is needed when interpreting the results. Moreover, the intervention used in this study was actually multifaceted (the use of the healthy plate plus group sessions). Even though part of the same group sessions was given to the control group, this intervention makes it difficult to determine the single effect of the use of healthy plate on weight changes. An additional study comparing the dietary intervention with the healthy plate with the dietary intervention without the healthy plate are needed to clarify these issues in the future.

## Conclusions

A lifestyle intervention program using a Japanese-style healthy plate, which was developed for portion control, may effectively reduce body weight in overweight and obese diabetic subjects in Japan. Further studies are needed to establish the efficacy of the healthy plate for the management of weight and weight-related disorders.
